# Thrombotic thrombocytopenic purpura treated with rituximab in systemic lupus erythematosus

**DOI:** 10.12861/jrip.2012.19

**Published:** 2012-09-01

**Authors:** Mansoor Karimifar

**Affiliations:** ^1^Department of Rheumatology, Isfahan University of Medical Sciences, Isfahan, Iran

**Keywords:** Thrombotic thrombocytopenic purpura, Rituximab, Systemic lupus erythematosus

**Implication for health policy/practice/research/medical education:**

In cases of refractory thrombotic thrombocytopenic purpura use of rituximab is appropriate.

Systemic lupus erythematosus (SLE) is a systemic disease that can lead to involvement of many organs such as bone marrow, blood vessels, skin, heart, kidneys and central nervous system (1). Thrombotic thrombocytopenic purpura (TTP) is considered as a serious complication of SLE (2-6). The patient, described in this paper, was a 16 year-old girl who has recently diagnosed with SLE and treated with 5 mg prednisone, 200 mg hydroxychloroquine, and 1000 mg calcium daily. After a few months, a thrombocytopenia of 40,000 /µl was detected. She was treated with 60 mg daily prednisolone and 25 gram of daily IV-IgG for 5 consecutive days, and finally, due to lack of response to treatment, she has been referred to Al-Zahra Medical Center. In clinical examination of peripheral blood, smear schistocyte of 8% and thrombocytopenia of 10,000/µl was detected. The value of serum LDH and hemoglobin were 1200 IU/ml and 9 g/dl, respectively. Following diagnosis of TTP, the patient underwent plasmapheresis. The patient with TTP were treated with daily plasmapheresis for 8 days replacing with 2 liters of fresh frozen plasma. In the meantime platelets reached to 140,000/µl and LDH regressed to normal value. One day after cessation of plasmapheresis, the platelet count felt to 60,000/µl, and in the second day it reached to 1000/µl. Also, hemoglobin dropped to 4g/dl and LDH raised to 1800 mg/dl. At this stage, two units of packed red blood cells infused and six additional plasmapheresis sessions were performed. However, there was not a significant improvement of platelet count. After ruling out of hepatitis B, 500 mg of rituximab once weakly and 125 mg methylprednisolone were intravenously infused, for three consecutive days. Then, 200 mg oral cyclosporine was started. Moreover plasmapheresis, continued again for 5 days and then stopped. Seven days later, the same dose of rituximab was repeated. At this time the platelet count was 105,000/µl, hemoglobin = 9 g/dl and LDH was normal and patient was discharged with 60 mg oral prednisolone, 200 mg cyclosporine and 200 mg hydroxychloroquine. Rituximab was repeated for two consecutive weeks after discharge. Finally, after four consecutive weeks of starting rituximab therapy, the platelet count was 260,000/µl, the hemoglobin reached to 11g/dl and, and the LDH remained normal.

## Conclusion

In cases of refractory thrombotic thrombocytopenic purpura use of rituximab is appropriate.

## Author’s contribution

MK is the single author of the manuscript.

## Conflict of interests

The author declared no competing interests.

## Ethical considerations

Ethical issues (including plagiarism, data fabrication, double publication) have been completely observed by the author.

## Funding/Support

None.

## References

[1] Bonakdar ZS, Mohtasham N, Karimifar M (2011). Evaluation of damage index and its association with risk factors in patients with systemic lupus erythematosus. J Res Med Sci.

[2] Kremer Hovinga JA, Vesely SK, Terrell DR, Lämmle B, George JN (2010). Survival and relapse in patients with thrombotic thrombocytopenic purpura. Blood.

[3] Fakhouri F, Vernant JP, Veyradier A, Wolf M, Kaplanski G, Binaut R (2005). Efficiency of curative and prophylactic treatment with rituximab in ADAMTS13-deficient thrombotic thrombocytopenic purpura: a study of 11 cases. Blood.

[4] Scully M, Cohen H, Cavenagh J, Benjamin S, Starke R, Killick S (2007). Remission in acute refractory and relapsing thrombotic thrombocytopenic purpura following rituximab is associated with a reduction in IgG antibodies to ADAMTS-13. Br J Haematol.

[5] Gutterman LA, Kloster B, Tsai HM (2002). Rituximab therapy for refractory thrombotic thrombocytopenic purpura. Blood Cells Mol Dis.

[6] Jasti S, Coyle T, Gentile T, Rosales L, Poiesz B (2008). Rituximab as an adjunct to plasma exchange in TTP: a report of 12 cases and review of literature. J ClinApher.

